# Evidence of Chikungunya Virus Disease in Pakistan Since 2015 With Patients Demonstrating Involvement of the Central Nervous System

**DOI:** 10.3389/fpubh.2018.00186

**Published:** 2018-07-10

**Authors:** Kelli L. Barr, Erum Khan, Joveria Q. Farooqi, Kehkashan Imtiaz, Dhani Prakoso, Faisal Malik, John A. Lednicky, Maureen T. Long

**Affiliations:** ^1^Department of Comparative, Diagnostic, and Population Medicine, University of Florida, Gainesville, FL, United States; ^2^Laboratory Medicine, Department of Pathology, Aga Khan University, Karachi, Pakistan

**Keywords:** Chikungunya, neutralization, neurological, encephalitis, arbovirus, West Nile virus, arthralgia

## Abstract

Several arboviruses are endemic to and co-circulate in Pakistan. In recent years, Pakistan has observed a rise in arboviral infections. A cross-sectional study for arboviral diseases, which included screening for Chikungunya virus (CHIKV), was initiated in 2015 to determine which pathogens were causing disease in patients presenting to health care services. Exposure to CHIKV was verified via detection of viral nucleic acids or virus-specific IgM with virus-specific neutralizing antibodies. Out of 997 enrolled patients presenting with clinical features suggestive of arboviral disease, 102 patients were positive for CHIKV IgM antibodies and 60 patients were positive for CHIKV nucleic acids or neutralizing antibodies. The data presented here show that CHIKV has been circulating in Pakistan since April of 2015. CHIKV infections were detected in study subjects up to the conclusion of our enrollment period in July 2017. Syndromic and clinical data show that arthralgia was associated with CHIKV as was rash, fever greater than 38°C, and lymphopenia. Neurological symptoms were reported in 49% of CHIKV suspect patients and in 46.6% of confirmed infections. Acute disseminated encephalomyelitis was diagnosed in 5% of confirmed infection and various manifestation of encephalitis diagnosed in an additional 16.6% of patients with confirmed CHIKV infections. CHIKV-exposed patients were just as likely to present with neurological symptoms and encephalitis as patients with West Nile Virus infections but were 4.57 times more likely to have lymphopenia. This proportion of neurological symptoms may be a complicating factor in countries where WNV and/or JEV co-circulate with CHIKV.

## Introduction

Chikungunya virus (CHIKV) is an enveloped, single-stranded, positive-sense RNA flavivirus that is maintained in nature between humans and *Aedes* mosquitos. These vectors are found throughout the world and are associated with a variety of viral outbreaks on all continents except Antarctica. In recent years, Pakistan has experienced significantly increased burdens of arboviral diseases including Dengue virus (DENV), West Nile virus (WNV), and Crimean Congo Hemorrhagic Fever (CCHF) (1–4). Unfortunately, reporting and surveillance for these diseases is limited due to the dissolution of the Pakistan Federal Ministry of Health in 2011.

To assist in the development of arbovirus diagnostic expertise, a Pakistan-US collaborative arbovirus surveillance program was initiated in the Sindh region of Pakistan in 2015 with the aim to identify which arboviruses were causing undifferentiated febrile illness in humans presenting to health care services (5). This study made use of instruments and procedures in accordance with CDC/WHO guidelines for viral diagnosis which require isolation of virus and/or viral nucleic acids or detection of virus specific IgM antibodies with virus-specific neutralizing antibodies from patient serum (6, 7). In addition, clinical and pathological data were collected.

Here we report upon the active and persistent circulation of CHIKV in humans in the southern region of Pakistan during 2015–2017. We also describe clinical and syndromic features of CHIKV infections in the region. Analysis of patient data revealed that neurological symptoms are present in a significant portion of CHIKV exposed patients.

## Methods

### Patient enrollment and case definitions

Patients presenting with clinical symptoms indicative of arboviral disease were enrolled under a cross-sectional, observational study from April 2015 to July 2017 (5). This study aimed to identify which arboviruses (DENV, WNV, Japanese Encephalitis virus (JEV), and CHIKV) were the cause of acute undifferentiated febrile illness in the Sindh region of Pakistan (5). Informed consent and study procedures were reviewed and approved by the Ethics Review Committee at Aga Khan University (#3183-PAT-ERC-14) and the Institutional Review Board at the University of Florida (#201500908). All enrolled subjects gave written consent in accordance with the Declaration of Helsinki.

Patients presenting with syndromic findings of arboviral disease were enrolled. Symptoms included: acute fever onset, headache, myalgia, arthralgia, rash, or gastrointestinal symptoms as stated in the CDC clinical description of arboviral illness (6). Since neuroinvasive arboviruses are endemic to Pakistan, patients presenting with symptoms indicative of neuroinvasive disease were also enrolled. These patients required syndromic findings of “acute onset of fever with headache, myalgia, stiff neck, altered mental status, seizures, limb weakness, or cerebrospinal fluid (CSF) pleocytosis” for enrollment (6). We also enrolled patients presenting with acute disseminated myeloencephalitis (ADEM), meningitis, encephalitis, acute flaccid paralysis, cranial nerve palsies, focal neurological deficits, or vertigo.

During December 2016 and January of 2017, we also enrolled patients presenting with clinical syndromes reflecting CDC/WHO case definitions for CHIKV which included “acute onset of fever, polyarthralgia, headache, maculopapular rash, myalgia, conjunctivitis, and nausea/vomiting (8, 9). This was done in order to assist the physicians at our study sites with the sudden influx of patients during a CHIKV outbreak. Patient data was double-entered into a commercial spreadsheet program (EXCEL, Microsoft, Redmond, WA).

Patients included males and females between 10 and 86 years age meeting the enrollment criteria on the day of enrollment. Patient samples were screened for malaria, influenza, CCHF, typhoid, and sepsis. Patients positive for these conditions were excluded from further testing or analysis. Patients were tested for DENV NS1 antigen using a manufactured kit (Panbio Dengue Early Rapid Test NS1 Antigen Capture Test, Alere, Waltham, MA) following the manufacturer's instructions. All NS1 negative patients and patients presenting with neurological syndromes that met the case criteria for neuroinvasive disease were evaluated for exposure to WNV and JEV via IgM capture ELISA (West Nile Detect, JE Detect Inbios, Seattle WA) and rt-PCR. Patients that were negative for DENV NS1 antigen as well as WNV and JEV IgM antibodies were then evaluated for CHIKV IgM antibodies using a commercial IgM ELISA kit (CHIKjj Detect, Inbios, Seattle WA).

### RT-PCR

CHIKV IgM positive patients and few select patients with severe polyarthralgia were screened for virus via rt-PCR. CHIKV primers and probes were constructed based on the NS2A gene (10). RNA was isolated from human serum samples using a commercial RNA extraction kit (QIAamp Viral RNA kit, QIAGEN, Valencia, CA) and reverse transcribed to cDNA using a commercial cDNA kit (iScript cDNA Synthesis Kit, Bio-Rad, Hercules, CA). PCR was performed using a commercial reaction mix (iTaq Universal Probe Supermix,Bio-Rad), 500 nM of forward and reverse primers and 175 nM of fluorogenic probe labeled at the 5′ end with FAM reporter dye and the 3′ end with a commercial quencher (BHQ-1, Biosearch Technologies, Petaluma, CA) in a 20 μl total reaction volume. Cycling conditions were as follows: 95°C for 2 min and 40 cycles of 95°C for 15 s and 60°C for 1 min.

### Plaque reduction neutralization test (PRNT)

Patient samples that were negative for CHIKV via rt-PCR were evaluated for CHIKV neutralizing antibodies via a plaque reduction neutralization test (PRNT). The viral strain, CHIKV 181/25 (WRCEVA) was used for all testing. Dilutions of patient serum (1:10 followed by 4-fold serial dilutions up to 1:160) were mixed with an equal volume of MEM containing ~50 plaque forming units (PFU) of virus and incubated for 1 h at 37°C. After incubation, this mixture was inoculated onto confluent Vero cells in 12-well plates, which were then incubated for an hour at 37°C. After incubation, the inoculum was removed and an overlay of MEM with 0.5% methylcellulose, 2.5% FBS, non-essential amino acids, penicillin, streptomycin, and amphotericin B was added to the wells and the plates returned to the incubator until plaques were visible. For counting, plaques were visualized by staining with a fresh mixture of 50% methanol, 43% ethanol, 7% acetic acid, and 0.1% wt/vol Coomassie blue. The antibody titer was determined as the highest dilution that neutralized at least 50% of the virus inoculum. A 50% reduction in plaques was set as the cut-off for a positive test in order to detect low positive responses in acute sera.

### Statistical analysis

Statistical analyses were performed on clinical data using a commercially available statistical software program (MedCalc 17.9.7). Logistic regression for dichotomous independent variables was performed. Symptoms and clinical features listed in CDC/WHO case definitions were set as the independent variables (6). Odds ratios (OR) were calculated with 95% confidence intervals. Ratios with a *p*-value < 0.05 were considered significant. Pearson's correlation coefficients and associated *p*-values were calculated to identify potential relationships between different variables in the data set. An ANOVA with a Tukey-Kramer *post-hoc* test was performed on non-dichotomous data. Chi-squared tests were used to verify normal distribution of the population. A subset of 14 gender and age matched patients enrolled in this study that were negative for CHIKV via rt-PCR, IgM ELISA, and PRNT were included as non-infected controls.

### Diagnostic criteria

Patients were confirmed positive for CHIKV exposure according to CDC criteria which requires “isolation of viral nucleic acid OR virus-specific IgM antibodies in serum with confirmatory virus-specific neutralizing antibodies in the same or a later specimen (6).” Thus, patients were classified as a CHIKV positive if they were positive via rt-PCR or if they had IgM antibodies via ELISA and a PRNT titer of at least 50% at any dilution. Patients that were positive for CHIKV IgM antibodies but negative for CHIKV rt-PCR and PRNT were classified as suspect since patient samples may have been obtained during the brief period when only IgM antibodies were present.

## Results

### Epidemiology

Patient enrollment for the seroprevalence study began in April of 2015. Over the course of 2 years, 997 patients with arboviral disease symptoms were enrolled. Of these, 638 were evaluated for CHIKV exposure via IgM ELISA. Of these 638 patients, 102 patients were IgM positive and from these, CHIVK was detected in 29 patient serum samples via rt-PCR and an additional 29 patients possessed neutralizing antibodies for CHIKV.

**Figure 1:**
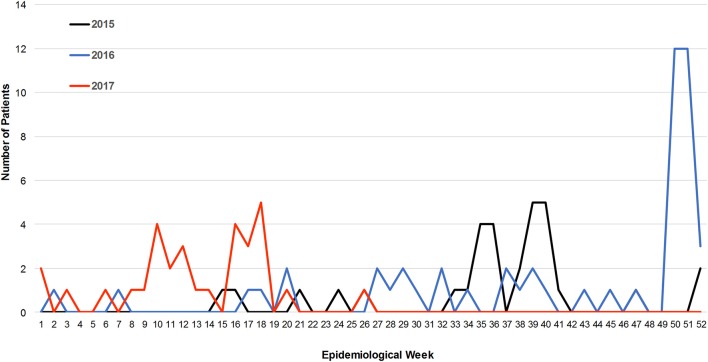
Incidence of CHIKV infections over a 27 month period. The number of IgM positive patients of CHIKV is plotted against epidemiological week.

Patients were confirmed positive for CHIKV exposure in all five study sites starting in April 2015 to the end of enrollment in July, 2017 with the majority of cases occurring prior to the recognized outbreak of December 2016 (Figure 1). The majority of cases were identified during the autumn and winter months (Figure 1). In 2015, low level, year-round activity was detected with peak activity occurring between late summer and autumn with smaller increases in winter months. During 2015, 28 cases of CHIKV were detected with 82% occurring between epidemiological week 32 and 45 (Figure 1). During 2016, 19 cases were detected during the same span of epidemiological weeks, consistent with 2015, but a large increase in patients (*n* = 24) testing IgM positive for CHIKV was detected beginning in epidemiological week 50 and continuing through the next 21 weeks (Figure 1). Pearson correlation coefficients were calculated to determine if there was any association between IgM ELISA results with PRNT titers. There was a very weak association between ELISA values and percent neutralization at the 1:10 serum dilution (*r* = 0.44). No other significant correlations were found for clinical data, occurrence of symptoms, and/or ORs.

### Patient characteristics

Based on diagnostic testing, 107 patients were positive for CHIKV via IgM, PRNT, and/or rt-PCR testing. Most of the patients were from Karachi. Patients categorized as positive for recent exposure included 54% that tested positive by IgM capture ELISA, 52% of those were also positive for CHIKV nucleic acids by rt-PCR, and 52% were positive by PRNT. Four patients were positive for CHIKV via rt-PCR and PRNT (Table S1). An additional five patients were negative for CHIKV IgM but positive via rt-PCR.

The mean age of CHIKV suspect patients was 33.6 years and the mean age of confirmed CHIKV patients was 37.6 years (Table S1). Age was a significant predictor for exposure (*p* = 0.048) for confirmed cases however, significance was lost when suspect cases were factored into the mean age (*p* = 0.977). Sixty-four percent of patients with confirmed CHIKV infection were males as were 57% of CHIKV suspect cases. Gender was not a significant predictor of CHIKV exposure.

### Clinical manifestations of CHIKV infection

#### Vital signs

Temperature, heart rate, respiratory rate, and blood pressure readings were recorded upon patient enrollment. The average heart rate was 98 beats per minute (bpm) for CHIKV suspect patients and 92 bpm for confirmed patients. A total of 35.3% of suspect patients and 23.3% of confirmed patients had heart rates greater than 100 bpm (Table 1). Average blood pressure readings were within normal limits and not a significant predictor of disease (Table 1). The average fever for CHIKV suspect patients was 38.3°C with an average duration of 5 days prior to hospital admission. The average fever of CHIKV confirmed patients was 37.9°C and a fever >38°C was a significant factor for CHIKV infection (*p* = 0.043). 64.7% of suspect patients and 38.3% of confirmed patients presented with fevers greater than 38°C (Table 1). For both suspect and confirmed patients, respirations were typically within normal limits and not associated with exposure though 4% of suspect and 5% of confirmed patients had respiratory rates greater than 25 breaths per minute (Table 1).

**Table 1:** Vital statistics for CHIKV suspect and confirmed patients.

	**CHIKV suspect**		**CHIKV confirmed**	
	**Mean (range)**	**OR (95% CI)**	**Mean (range)**	**OR (95% CI)**
	**N with abnormal values (%)**		**N with abnormal values (%)**	
Heart Rate (bpm)	98 (42–155)	1.55 (0.42–5.69)	92 (12–127)	1.4 (0.37–5.3)
	18 (35.3%)		14 (23.3%)	
Systolic (mmHg)	118 (80–195)	176E+006	124 (81–230)	
	3 (8.3%)		5 (15%)	569E+006
Diastolic (mmHg)	69 (51–92)		71 (50–100)	
	2 (5.5%)		2 (6%)	
Temperature (°C)	38.3 (36.2–40)	2.93 (0.86–9.95)	37.8 (36–40.5)	1.4 (0.41–4.71)
	33 (64.7%)		23 (38.3%)	
Respiration (bpm)	20.25 (13–28)	0.16 (0.02–1.12)	20.89 (12–34)	0.21 (0.03–1.41)
	2 (4%)		3 (5%)	

*Odds Ratios were calculated using a cohort of gender and age matched study patients that were negative for CHIKV*.

#### Clinocopathological testing

Measurements for hepatic enzymes, lymphocytes, platelets, and hemoglobin were recorded upon enrollment. In CHIKV exposed patients, 83.3% suspect and 58.3% confirmed patients tested for abnormally high levels of aspartate aminotransferase (Table 2). 52.3% of suspect patients and 18.7% of confirmed patients produced abnormal levels of alanine aminotransferase (Table 2). Abnormal levels of alanine aminotransferase were significantly less likely to occur for CHIKV confirmed patients than CHIKV negative patients (Table 2). Platelet counts for both suspect and confirmed CHIKV exposure patients were typically normal with 34.3% of suspect and 9.8% of confirmed patients exhibiting platelet counts indicative of either thrombocytopenia or thrombocytosis (Table 2). Five of seven patients with thrombocytosis also had neutrophil counts above normal (data not shown). Both suspect and confirmed patients were far less likely to exhibit thrombocytopenia than CHIKV negative patients (Table 2). Over 40% of CHIKV suspect and confirmed patients exhibited lymphopenia and CHIKV confirmed patients were 2.15 times more likely to have lymphopenia than CHIKV-negative patients (Table 2). Hemoglobin levels were below normal for 42.5% of suspect and 40% of confirmed patients but were no more likely to occur than for CHIKV negative patients (Table 2).

**Table 2:** Clinical testing for CHIKV suspect and confirmed patients.

	**CHIKV suspect**		**CHIKV confirmed**	
	**Mean (range)**	**OR (95% CI)**	**Mean (range)**	**OR (95% CI)**
	**N with abnormal values (%)**		**N with abnormal values (%)**	
Aspartate aminotransferase (IU/L)	96.6 (16–172)	0.17 (0.02–1.23)	53.7 (16–103)	0.19 (0.03–1.43)
	10 (83.3%)		7 (58.3%)	
Alanine aminotransferase (IU/L)	59.9 (10–119)	0.20 (0.01–2.89)	40.5 (18–96)	**0.13 (0.02–0.78)**
	11 (52.3%)		3 (18.7%)	
Total Lymphocyte Count (10^9^/L)	20.1 (4.3–40.4)	1.8 (0.52–6.20)	23.2 (2.7–58.5)	2.15 (0.64–7.21)
	23 (43%)		32 (42%)	
Platelet Count (10^9^/L)	236 (20–957)	**0.13 (0.03–0.56)**	269 (19–629)	**0.05(0.01–0.23)**
	15 (34.3%)		10 (9.8%)	
Hemoglobin (g/dL)	12.2 (7.1–18.1)	1.8 (0.52–6.20)	12.4 (7.0–16.3)	0.97 (0.29–3.30)
	23 (42.5%)		22 (40%)	

*Odds ratios were calculated using a cohort of gender and age matched study patients that were negative for CHIKV. Bold font indicates statistical significance*.

#### Symptoms

Clinical findings at the time of presentation for both suspect and confirmed CHIKV exposed patients included headache, fever, rash, myalgia, arthralgia, eye pain, and gastrointestinal symptoms (Table 3). Reporting of arthralgia occurred in 65% and 33.3% of confirmed and suspect patients, respectively, and overall CHIKV exposed patients were 3.06–12.67 times to exhibit joint pain (Table 3). Myalgia occurred in 45% of confirmed patients and 53% of suspect patients (Table 3). Reporting of a petechial or maculopapular rash occurred in 21.6% and 13.7% of confirmed and suspect patients, respectively, with these same patients having ORs 3.95 and 2.13 for this clinical finding (Table 3). Hemorrhage occurred in 5% and 4% of confirmed and suspect patients, respectively (Table 3). Gastro-intestinal disturbances occurred in 53.3% and 39.2% of CHIKV confirmed and suspect patients, respectively (Table 3). Eye pain occurred in 13.3% and 23.5% of suspect and confirmed, respectively, while headache occurred in 45% and 51% of patients in these classifications (Table 3). Respiratory symptoms consisting mainly of shortness of breath and/or difficulty breathing occurred in 13.3% and 8% of confirmed and suspect patients, respectively (Table 3). Urinary discomfort including burning micturition occurred in 6.6% and 11.8% of confirmed patients and suspect patients (Table 3). No significant association of positive CHIKV status was detected for headache, myalgia, eye pain, hemorrhage, gastrointestinal, respiratory, or urinary symptoms (Table 3).

**Table 3:** Incidence of symptoms in patients with chikungunya infection for suspect and confirmed cases of exposure.

**Status**	**CHIKV confirmed (*n*)**	**CHIKV IgM positive–(*n*)**	**CHIKV confirmed OR (95% CI)**	**CHIKV IgM positive OR (95% CI)**
Eye pain	5.5% (8)	23.5% (12)	448E+006	514E+006
Hemorrhage	5% (3)	4% (2)	0.49 (0.04–5.81)	0.84 (0.08–8.71)
Rash	21.6% (13)	13.7% (7)	3.95 (0.47–32.97)	2.13 (0.24–18.83)
Gastrointestinal	53.3% (32)	39.2% (20)	1.31 (0.42–4.1)	0.75 (0.23–2.37)
Headache	45% (27)	51% (26)	0.96 (0.31–3.0)	1.04 (0.35–3.47)
Myalgia	45% (27)	53% (27)	0.56 (0.18–1.78)	0.69 (0.21–2.22)
Arthralgia	65% (39)	33.3% (17)	**12.67 (2.6–61.74)**	**3.06 (0.62–15.15)**
Altered mental status	16.6% (10)	6% (3)	0.82 (0.19–3.43)	0.24 (0.0.4–1.34)
Seizures	10% (6)	8% (4)	1.58 (0.18–14.27)	1.14 (0.12–11.06)
Weakness	6.6% (4)	8% (4)	153E+006	158E+006
Encephalitis	16.6% (10)	15.7% (8)	1.17 (0.22–6.08)	1.32 (0.25–6.94)
Respiratory	13.3% (8)	8% (4)	442E+006	162E+006
Urinary	6.6% (4)	11.8% (6)	1.03 (0.11–10.03)	1.79 (0.2–16.12)
ADEM	5% (3)	15.7% (8)	150E+006	468E+006
Vertigo	3.3% (2)	11.8% (6)	54.2E+006	449E+006
Any neurological symptoms present	**46.6% (28)**	**49% (25)**	**5.48 (1.13–26.48)**	**6.25 (1.28–30.48)**

*Odds ratios were calculated using a cohort of gender and age matched study patients that were negative for CHIKV. Bold font indicates statistical significance*.

Symptoms of central nervous system (CNS) involvement occurred in 49% of and 46.6% CHIKV suspect and confirmed patients, respectively (Table 3). These symptoms included vertigo, altered mental status, seizures, ADEM, and encephalitis. Suspect patients were 6.25 times more likely to present with neurological symptoms than the patients testing negative for CHIKV (Table 3). Likewise, CHIKV confirmed individuals were 5.48 times more likely to present with any of the above neurological symptoms than negative patients (Table 3). Altered mental status (i.e., confusion, disorientation, irritability, drowsiness, and restlessness) was reported in 16.6% and 6% of confirmed and suspect patients (Table 3). Seizures were reported in 10% and 8% of confirmed and suspect patients (Table 3) Encephalitis, including meningoencephalitis and meningitis, was reported in 16.6% and 15.7% confirmed and suspect patients (Table 3). ADEM was reported for 5% of confirmed patients and for 15.7% of suspect patients (Table 3). Vertigo was seen in 3.3% and 11.8% of confirmed and suspect patients (Table 3).

**Figure 2:**
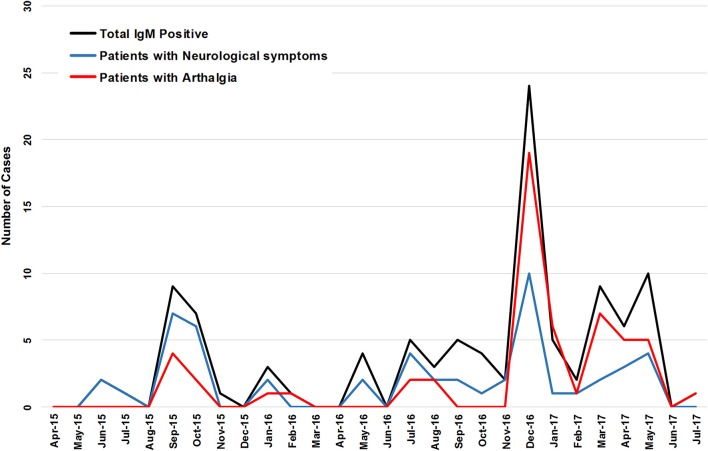
The incidence of CHIKV IgM+ patients and the reported incidence of arthralgia and neurological symptoms of a 27 month period.

No correlation was detected between the presence of arthralgia with neurological symptoms (*r* = −0.07629), or encephalitis (*r* = −0.1344), or seizures (*r* = −0.1645). Of 93 IgM positive patients (suspect and confirmed) presenting with CNS symptoms, 14 had concomitant arthralgia. Four of 18 patients with encephalitis had arthralgia. None of the patients with seizures had arthralgia. August through November 2015 saw 15 of 23 patients with neurological symptoms and 7 patients with arthralgia (Figure 2). Neurological symptoms were reported in the majority CHIKV-IgM+ patients prior to December 2016 whereas arthralgia was reported in no more than half of the same patients (Figure 2). Following the identification of CHIKV in December 2016, arthralgia was reported for nearly all patients (*p* = 0.0082) (Figure 2).

#### Syndrome comparison

The CHIKV confirmed cohort was compared with a WNV confirmed cohort to determine if any neurological symptoms were more likely to be present for either virus (1). Symptoms of CNS involvement included ADEM, encephalitis, vertigo, altered mental status, seizures, and weakness. Odds ratios ranged from 0.40 to 1.36 for all symptoms indicating no significant association for either virus for any symptom (Table 4). It was of interest to note that patients with CHIKV infections were 4.57 times more likely to present with lymphopenia than patients with WNV infections (Table 4). While over half of CHIKV infected patients exhibited abnormally low lymphocyte counts, only 20.3% of WNV infected patients had low lymphocytes (Table 4).

**Table 4:** Incidence of neurological symptoms in CHIKV and WNV confirmed patients.

**Symptom**	**CHIKV (*n*)**	**WNV (*n*)**	**OR (95% CI)**
Headache	45% (27)	42.3% (25)	1.00 (0.48–2.07)
Altered mental status	19.6% (10)	28.8% (17)	0.44 (0.17–1.10)
Seizures	10% (6)	15.2% (9)	0.51 (0.16–1.64)
Weakness	6.7% (4)	5% (3)	1.36 (0.29–6.35)
Encephalitis	19.6% (10)	13.5% (8)	1.3 (0.47–3.57)
ADEM	5% (3)	11.8% (7)	0.40 (0.1–1.62)
Vertigo	3.3% (2)	5% (3)	0.65 (0.11–4.07)
Amy neurological symptoms present	46.6% (28)	49.1% (29)	0.87 (0.42–1.8)
Lymphopenia	53.3% (32)	20.3% (12)	**4.57 (2.0–10.4)**

*Bold font indicates statistical significance*.

## Discussion

This study demonstrates that CHIKV has been circulating in Pakistan since 2015; at least 18 months prior to its recognized emergence during the reported outbreak in December 2016 in Karachi, Pakistan. The data also show that neurological symptoms are often present in CHIKV-infected patients presenting with undifferentiated fever. This may be a complicating factor in countries where other neuroinvasive arboviruses co-circulate such as WNV and JEV. In areas were viruses co-circulate, syndromic presentation can influence diagnosis, clinical investigations, and patient care.

Based on their neurological symptoms, patients enrolled prior to the 2016 CHIKV outbreak were routed for diagnostics for WNV and JEV, which are known to cause neuroinvasive disease and are endemic in the region. These patients were ultimately negative for WNV and JEV IgM and rt-PCR and were classified as “indeterminate exposure.” In light of the 2016 outbreak, these patients were retrospectively evaluated for CHIKV exposure. The data show that, prior to the 2016 outbreak, 52% of CHIKV exposed persons exhibited neurological symptoms and only 23% of patients reported arthralgia. During and after the outbreak, 64% of patients reported arthralgia and 40% reported neurological symptoms however, few patients reported both symptoms. Whether this was due to a function of CHIKV awareness or a different strain of CHIKV is unknown. CHIKV was not suspected, especially prior to the outbreak, due to the lack of arthralgia in most patients. While neurological syndromes are not usually reported for CHIKV outbreaks (11–13), encephalitis and other severe neurological manifestations have been reported recently (14–16).

The data presented here were not collected with the objective of specifically identifying CHIKV. Most arboviruses of humans have overlapping syndromes and these viruses often exhibit new symptoms when emerging in new locales. Thus, any patient meeting the case definition of arboviral disease was tested for exposure to several arboviruses. As a result, the incidence of clinical manifestation for the population presented here are unique from what has been described elsewhere for this outbreak (17–20). For instance, arthralgia is accepted as the defining symptom of CHIKV infection and is often used as enrollment criteria in most patient-based studies (21, 22). This is evident in recent studies that report over 90% of patients exhibiting arthralgia, both in Pakistan and the Western Hemisphere (17, 22, 23). Conversely, non-syndromic enrollment has reported an incidence of the clinical manifestations of CHIKV that reflect what is reported here (24). The data herein suggest that neurological disease is a likely manifestation of CHIKV infection as is the case for many other alphaviruses pathogenic to humans.

Commercial IgM capture ELISA formats are the cornerstone for detection of acute exposure to arboviruses. In areas where closely related viruses co-circulate, cross-reactivity is a significant problem and is the reason why the detection of virus specific neutralizing antibodies or nucleic acid is required for a definitive diagnosis (6, 7, 25, 26). In Pakistan, Sindbis virus (SINV) is the only alphavirus known to circulate in the region which could confound test results. While disease due to SINV has not been reported in Pakistan, evidence of seropositive people was reported in 1983 (27). Interestingly, both CHIKV and SINV were detected with similar frequencies; around 1–1.5% of 43 humans. While SINV has been detected in many countries, actual human disease is limited and outbreaks are often confined to the same locales, and almost exclusively in northern Europe. The patients reported in this study were defined by their clinical case criteria and CHIKV nucleic acids were detected in many patients concurrent with patients with antibody only evidence of CHIKV exposure. Notably, the rash associated with SINV is pruritic and skin biopsies are often positive for virus. Pruritic rash was not reported in any of our patients.

For this study, a commercial IgM ELISA kit was used to identify CHIKV suspect cases. While some diagnostics and data sets are based on the presence of virus-specific neutralizing antibodies, this may not correlate with acute exposure. A positive PRNT with a negative IgM indicates historical exposure of at least 4–6 months. Interpretation of PRNT testing alone must rely on paired serum. Furthermore, neutralizing antibodies are also highly cross-reactive with other closely related viruses thus, neutralizing thresholds are not calculated below 50% reduction of viral plaques (25, 26). Serum from 55 patients with IgM antibodies to CHIKV were evaluated for the presence of virus-specific neutralizing antibodies via PRNT. When the neutralizing threshold for this data was set at 80% neutralization, 14 patients (25.4%) were interpreted as positive for CHIKV exposure (Table S1). Since IgM positive patients would not be expected to have a robust neutralizing response, a 50% neutralization threshold was used which is consistent with WHO diagnostic guidelines (7). Under these parameters, 29 (57.7%) of patients were interpreted as positive for CHIKV exposure (Table S1).

In conclusion, CHIKV has been circulating in Pakistan since 2015 and our analysis indicates CNS symptomology in over 40% of patients. This proportion of neurological symptoms may be resultant from the broader enrollment strategy based on increased body temperature without respect to other clinical abnormalities. CHIKV should be considered in patients presenting with febrile disease irrespective of the presence of acute arthritis. When multiple arboviruses co-circulate during high seasonal activity, testing for multiple pathogens should be performed to differentiate arbovirus etiological agents. Accurate diagnosis of arboviral infections instructs the healthcare industry of circulating viruses and can have a direct effect on mosquito mitigation efforts since different vectors are involved.

## Author contributions

KB, EK, ML, and JL conceived and designed the experiments. KB, DP, JF, KI, and FM performed experiments and collected data. KB, ML, EK, JF, KI, and DP analyzed data and interpreted results. All authors contributed to drafting and editing the manuscript.

### Conflict of interest statement

The authors declare that the research was conducted in the absence of any commercial or financial relationships that could be construed as a potential conflict of interest.

## Supplementary material

Table S1 Serological testing of patients with suspect chikungunya infections. Data are presented for IgM ELISA, rt-PCR, and PRNT analysis of patient serum. According to the manufacturer, ISR values of 1.00 or greater are positive for CHIKV IgM antibodies. ^*^ISR, Immune Status Ratio. This represents the ratio of the patient serum OD reading with the control serum reading.
