# Supplementary material for: Evidence of Chikungunya Virus Disease in Pakistan Since 2015 With Patients Demonstrating Involvement of the Central Nervous System
Source: Front Public Health. 2018 Jul 10;6:186. doi: 10.3389/fpubh.2018.00186 (PMC6048291; doi:10.3389/fpubh.2018.00186)
Supplement: Table S1 — Serological testing of patients with suspect chikungunya infections. Data are presented for IgM ELISA, rt-PCR, and PRNT analysis of patient serum. According to the manufacturer, ISR values of 1.00 or greater are positive for CHIKV IgM antibodies. *ISR, Immune Status Ratio. This represents the ratio of the patient serum OD reading with the control serum reading. [file Table_1.DOCX]

| Patient ID | ISR | rt-PCR | 1:10 | 1:40 | | 1:160 |
| --- | --- | --- | --- | --- | --- | --- |
| H-020-15 | 1.05 |  |  |  | |  |
| H-035-15 | 1 |  |  |  | |  |
| H-038-15 | 1.02 |  |  |  | |  |
| H-071-15 | 1.11 |  |  |  | |  |
| H-074-15 | 1.14 |  |  |  | |  |
| H-090-15 | 2.12 |  | 65.28 | 40.28 | | 0.00 |
| H-176-16 | 1.05 |  |  |  | |  |
| H-178-16 | 1.18 |  |  |  | |  |
| H-182-16 | 1.04 |  | 72.92 | 58.33 | | 0.00 |
| **H-63-15** | 1.00 |  |  |  | |  |
| **H-70-15** | 1.34 |  |  |  | |  |
| K-005-15 | 1.01 |  |  |  | |  |
| K-022-15 | 1 |  |  |  | |  |
| K-037-15 | 1.11 |  |  |  | |  |
| **K-06-15** | 1.2 |  |  |  | |  |
| K-077-15 | 1.03 |  |  |  | |  |
| K-088-15 | 1.07 |  |  |  | |  |
| **K-123-15** | 1.06 |  |  |  | |  |
| **K-124-15** | 1.25 |  |  |  | |  |
| **K-140-15** | 1.02 |  |  |  | |  |
| K-146-15 | 1.15 |  |  |  | |  |
| K-231-16 | 1.38 |  | 62.50 | 72.22 | | 46.53 |
| K-233-16 | 1.54 |  | 84.72 | 74.31 | | 62.50 |
| K-237-16 | 1.40 |  |  |  | |  |
| K-238-16 | 1.37 |  | 62.50 | 59.72 | | 45.14 |
| K-249-16 | 1.41 |  |  |  | |  |
| K-251-16 | 1.46 |  | 78.47 | 63.89 | | 55.56 |
| K-256-16 | 1.40 |  |  |  | |  |
| K-260-16 | 1.79 |  |  |  | |  |
| K-267-16 | 2.74 |  |  |  | |  |
| K-295-16 | 1.57 |  | 75.00 | 61.11 | | -1.39 |
| K-300-16 | 1.50 |  |  |  | |  |
| K-314-16 | 1.34 |  |  |  | |  |
| K-324-16 | 4.06 |  |  |  | |  |
| K-327-16 | 1.41 |  |  |  | |  |
| K-334-16 | 1.63 |  |  |  | |  |
| K-362-16 | 1.44 |  | 77.08 | 79.17 | | 43.06 |
| K-374-16 | 1.40 |  |  |  | |  |
| K-381-16 | 6.65 |  | 97.92 | 93.75 | | 80.56 |
| K-391-16 | 0.74 | + |  |  | |  |
| K-392-16 | 0.43 | + |  |  | |  |
| K-394-16 | 11.75 |  |  |  | |  |
| K-395-16 | 1.51 |  |  |  | |  |
| K-396-16 | 11.80 | + |  |  | |  |
| K-397-16 | 1.92 |  |  |  | |  |
| K-398-16 | 1.03 | + |  |  | |  |
| K-399-16 | 1.71 | + |  |  | |  |
| K-400-16 | 3.13 | + |  |  | |  |
| K-412-17 | 0.22 | + |  |  | |  |
| K-413-17 | 3.51 |  |  |  | |  |
| K-415-17 | 3.78 |  |  |  | |  |
| K-416-17 | 0.25 | + |  |  | |  |
| K-417-17 | 0.25 | + |  |  | |  |
| K-418-17 | 0.33 | + |  |  | |  |
| K-419-17 | 13.10 |  |  |  | |  |
| K-421-17 | 8.41 |  |  |  | |  |
| K-422-17 | 0.30 | + |  |  | |  |
| K-423-17 | 7.14 |  |  |  | |  |
| K-425-17 | 6.65 | + |  |  | |  |
| K-426-17 | 9.32 |  |  |  | |  |
| K-430-17 | 9.24 |  |  |  | |  |
| K-431-17 | 0.32 | + |  |  | |  |
| K-432-17 | 0.28 | + |  |  | |  |
| K-433-17 | 2.44 |  |  |  | |  |
| K-434-17 | 0.29 |  | 75.69 | 58.33 | | 54.86 |
| **K-435-17** | 0.31 | + |  |  | |  |
| **K-437-17** | 3.58 |  |  |  | |  |
| K-441-17 | 8.69 |  |  |  | |  |
| K-443-17 | 5.85 |  |  |  | |  |
| **K-444-17** |  | + |  |  | |  |
| K-445-17 | 5.46 |  |  |  | |  |
| **K-447-17** |  | + |  |  | |  |
| **K-451-17** |  | + |  |  | |  |
| **K-469-17** |  | + |  |  | |  |
| K-901-16 | 1.78 |  | 57.64 | 56.25 | | 61.11 |
| K-904-16 | 1.30 |  | 63.89 | 79.17 | | 67.36 |
| K-905-16 | 7.02 |  | 95.83 | 96.53 | | 84.03 |
| K-906-16 | 10.20 |  | 100.00 | 100.00 | | 98.61 |
| K-907-16 | 0.69 | + |  |  | |  |
| K-909-16 | 8.40 |  | 98.61 | 97.22 | | 84.03 |
| K-910-16 | 10.30 |  | 100.00 | 98.61 | | 97.92 |
| K-912-16 | 1.78 | + |  |  | |  |
| K-913-16 | 11.65 |  | 100.00 | 100.00 | | 98.61 |
| K-914-16 | 11.51 |  | 100.00 | 100.00 | | 100.00 |
| K-915-16 | 1.40 | + |  |  | |  |
| K-916-16 | 1.28 | + |  |  | |  |
| K-917-16 | 1.20 | + |  |  | |  |
| K-918-16 | 1.15 |  | 84.03 | 70.14 | | 76.39 |
| K-919-16 | 11.36 |  | 97.92 | 95.83 | | 88.89 |
| K-920-16 | 11.58 |  | 97.92 | 87.50 | | 84.03 |
| K-921-16 | 1.80 |  | 65.28 | 66.67 | | 27.08 |
| K-922-16 | 1.92 | + |  |  | |  |
| K-923-17 | 0.16 | + |  |  | |  |
| K-925-17 | 2.27 |  | 97.92 | 93.06 | | 91.67 |
| K-926-17 | 0.41 | + |  |  | |  |
| K-927-17 | 1.13 |  | 93.75 | 88.19 | | 82.64 |
| K-929-17 | 1.57 |  | 99.31 | 100.00 | | 98.61 |
| K-932-17 | 1.57 | + |  |  | |  |
| K-937-17 | 3.41 |  |  |  | |  |
| L-008-15 | 1.02 |  | 59.72 | 43.75 | | 25.69 |
| L-052-16 | 0.98 |  |  |  | |  |
| M-043-15 | 1.03 |  |  |  | |  |
| M-105-16 | 1.1 |  |  |  | |  |
| **M-57-15** | 1.06 |  |  |  | |  |
| S-014-15 | 1.1 |  |  |  | |  |
| S-016-15 | 1.21 |  |  |  | |  |
| S-017-15 | 1.12 |  |  |  | |  |
| S-021-15 | 1.01 |  |  |  | |  |
| S-025-15 | 1 |  |  |  | |  |
| S-039-15 | 4.56 | + | 75.00 | 50.00 | | 50.00 |
| S-052-15 | 1.13 | + |  |  | |  |
| **S-35-15** | 1.11 |  |  |  | |  |
| Control Serum | | | | | | |
| H-082-15 | 0.75 | - | 0.00 | | 0.00 | 0.00 |
| K-087-15 | 0.80 | - | 0.00 | | 0.00 | 0.00 |
| K-074-15 | 0.82 | - | 0.00 | | 0.00 | 0.00 |
| K-035-15 | 0.70 | - | 0.00 | | 0.00 | 0.00 |
| K-121-15 | 0.86 | - | 0.00 | | 0.00 | 0.00 |
| K-139-15 | 0.80 | - | 0.00 | | 0.00 | 0.00 |
| K-140-15 | 0.76 | - | 0.00 | | 0.00 | 0.00 |
| K-153-15 | 0.51 | - | 0.00 | | 0.00 | 0.00 |
| K-006-15 | 0.88 | - | 0.00 | | 0.00 | 0.00 |
| K-005-15 | 0.87 | - | 0.00 | | 0.00 | 0.00 |
| K-056-15 | 0.86 | - | 0.00 | | 0.00 | 0.00 |
| L-033-15 | 0.51 | - | 0.00 | | 0.00 | 0.00 |
| K-155-15 | 0.57 | - | 0.00 | | 0.00 | 0.00 |
| K-174-15 | 0.42 | - | 0.00 | | 0.00 | 0.00 |
| K-008-15 | 0.83 | - | 0.00 | | 0.00 | 0.00 |
| K-058-15 | 0.59 | - | 0.00 | | 0.00 | 0.00 |
